# Physical Activity Interventions for Colorectal Cancer Survivors: A Systematic Review and Meta-analysis of Randomized Controlled Trials

**DOI:** 10.1097/NCC.0000000000000888

**Published:** 2020-09-14

**Authors:** Youjin Jung, Joohyun Chung, Heesook Son

**Affiliations:** Author Affiliations: National Evidence-Based Healthcare Collaborating Agency, Seoul, South Korea (Ms Jung); College of Nursing, University of Massachusetts at Amherst (Dr Chung); Chung-Ang University, Red Cross College of Nursing, Seoul, South Korea (Dr Son).

**Keywords:** Colorectal cancer survivors, Physical activity, Quality of life

## Abstract

**Background:**

Physical activity (PA) has been shown to improve total mortality and colorectal-specific mortality risk; however, colorectal cancer (CRC) survivors have lower rates of PA compared with survivors with other types of cancers.

**Objective:**

To examine the effect of PA interventions on CRC survivors.

**Methods:**

A systematic review and meta-analysis were conducted to identify randomized controlled trials that met the inclusion criteria, which included an intervention designed to increase PA and more than 1 outcome of interest. Random effects of the meta-analyses were performed using Review Manager 5.3.

**Results:**

Eight publications representing 7 randomized controlled trials of 803 participants were identified. All studies used a combination of behavioral change methods. Physical activity interventions significantly improved disease-specific quality of life, PA level, and maximum amount of oxygen and did not show significant improvements for fatigue and body mass index among CRC survivors.

**Conclusions:**

We provided evidence that PA interventions were effective in improving disease-specific quality of life, PA level, and maximum amount of oxygen; however, they did not improve fatigue and body mass index. Further randomized controlled trials are needed to determine the optimal mode of delivering PA intervention for CRC survivors.

**Implications for Practice:**

As the survival rate of patients with CRC increases, survivors of CRC need to increase PA in a community setting after completing primary treatments. Effective and efficient modes of PA intervention delivery could improve health-related outcomes and address specific barriers for CRC survivors.

As a commonly diagnosed cancer in both men and women, colorectal cancer (CRC) represents a global concern^1^; it is the third leading cause of death worldwide and one of the 3 most prevalent cancers in Korea.^2,3^ Owing to advancements in treatments and early detection from routine screening, the survival rate of CRC has increased in recent years such that the 5*-*year relative survival rate is 89.9% to 71.3% in localized and regional stages, respectively,^4^ suggesting that the individuals live longer with cancer as those with other chronic diseases.

A cancer survivor is any individual with cancer from the time of initial diagnosis to the end of life,^5^ encompassing those who have been newly diagnosed, have completed their primary treatment, and have no evidence of active disease.^6^ When considering the phenomena of late- and long-term effects of the primary treatment of cancer on the health and well-being of survivors,^7,8^ the posttreatment stage of survivorship may be a critical period to practice self-care for the management of the disease. In particular, CRC survivors need to maintain a healthy lifestyle during the posttreatment period as physical activity (PA) reduces the risk of cancer recurrence and mortality after postoperative adjuvant treatment for CRC survivors.^9^

Physical activity is critical for cancer survivors.^10^ The 2018 PA guidelines for cancer survivors recommend that survivors should avoid inactivity by engaging in PA for at least 150 to 300 minutes per week of moderate aerobic activity, or 75 to 150 minutes per week of vigorous activity (or an equivalent combination of moderate- and vigorous-intensity aerobic activity) and at least 2 days per week of strength activity to gain substantial health benefits.^11–14^ However, adherence to PA guidelines has been shown to be lacking among cancer survivors, with approximately 44% of long-term cancer survivors not meeting the guidelines.^15^ In particular, CRC survivors have been found to have the lowest rates of PA when compared with survivors of other types of cancer^16^ although PA after diagnosis has been associated with lowering the overall risk of mortality, as well as CRC-specific mortality.^17^

A variety of interventions including the utilization of telephone-based multimodal health behaviors,^18^ a wearable device,^19^ and a mobile healthcare application^20^ have been found to improve PA for CRC survivors. Systematic reviews and meta-analyses have revealed that interventions may improve patient outcomes, including their quality of life, physical functioning, and level of anxiety.^21–23^ However, limited evidence regarding the effects of PA on the health outcomes of CRC survivors has been reported, as most studies have included samples of patients with different cancer diagnoses.^24,25^ Thus, the unique effect of PA on CRC patients remains unclear.

In recent years, Cramer and colleagues^26^ examined the effectiveness of exercise for CRC patients, whereas Brandenbarg et al^27^ studied the effect of PA on fatigue. However, these studies included CRC patients who were receiving primary and adjuvant treatments. Because the aims and the content differ between the interventions for CRC patients actively receiving treatment and those who have completed treatment, aggregating these different populations in a single study may obfuscate the efficacy of the intervention. Furthermore, Eyl and colleagues^28^ investigated the association between PA and quality of life in CRC survivors who were 5 or more years postdiagnosis; however, the authors included both observational and cross-sectional studies in the 7 selected studies, which limited the conclusions regarding the effects of PA. There is evidence of PA’s benefits for survivors of breast cancer and prostate cancer; however, research among other cancer survivor groups has been relatively understudied.^29^ Therefore, this study aimed to examine the effect of PA interventions for CRC survivors.

## Methods

### Design

We conducted a systematic review and meta-analysis to examine the effect of randomized controlled trials that aimed to increase PA among CRC survivors. We followed the *Cochrane Handbook for Systematic Review of Interventions*,^30^ as well as PRISMA (Preferred Reporting Items for Systematic Reviews and Meta-analyses) guidelines.^31^

### Key Questions

The key questions for our systematic review were developed using PICO-SD guidelines (ie, patient, intervention, comparison, outcome, study design). For inclusion in this review, the sample of patients was required to be CRC survivors. We did not limit the cancer stage and treatment methods for patients; however, we included only studies of CRC survivors who had completed the primary treatment (ie, chemotherapy or radiation) prior to their recruitment. The studies included interventions that encompassed a variety of programs that used solely PA interventions, as well as those interventions that included mixed or comprehensive programs to promote lifestyle changes. Studies were eligible for inclusion if the comparison group was composed of CRC survivors receiving usual care and no intervention. The outcome variables of interest were the patients’ quality of life, level of PA, fatigue, Vo_2max_ (ie, the maximum amount of oxygen a person can utilize during PA),^32^ and their body mass index (BMI). Finally, we included only studies that were randomized controlled trials.

### Search Strategy

A search strategy was developed using an iterative process based on the recommendations of the Cochrane Collaboration.^30^ We searched for studies that compared a PA intervention with usual care in samples of CRC survivor patients using Ovid-MEDLINE, Ovid-EMBASE, Cochrane Central Register of Controlled Trials, and Cumulative Index to Nursing and Allied Health Literature until February 4, 2019. Five Korean databases (KoreaMed, KMbase, KISS, RISS, and NDSL) were also used without restrictions in publication year or language. Finally, we conducted manual searches through the reference lists of identified articles to locate additional potential studies.

The selected search terms included keywords as well as using Medical Subject Heading (MeSH) terms that specified participants and intervention. Search terms for patients were “exp Colorectal Neoplasms/,” “((colorectal$ or colon$ or rect$) adj2 (cancer$ or neoplasm$ or tumor$ or tumor$ or malignan$ or carcinoma$ or adenocarcinom$)).tw.”. Search terms for intervention were “physical activit$.tw.,” “exercis$.tw.,” “(life style or life style).tw.,” “exp Sports/,” “walking/,” “running/,” “jogging,” and other related terms. Details of the search strategy are provided in the Appendix 1.

### Study Selection

#### INCLUSION AND EXCLUSION CRITERIA

Studies were eligible for inclusion in the review and meta-analysis if they (*a*) evaluated an intervention designed to increase PA, including trials focusing on multiple behaviors; (*b*) included adult CRC patients who had completed treatment; (*c*) reported at least one of the outcomes of interest; and (*d*) were randomized controlled trials. Studies were excluded if they (*a*) did not include an appropriate comparison group, such as a comparison of high-dose intervention with low-dose intervention; (*b*) were only available as an abstract; (*c*) were written as a review, editorial, or commentaries, rather than a randomized controlled trial; and (*d*) were published in a language other than Korean or English.

#### IDENTIFICATION AND SELECTION OF STUDIES

After all of the potentially relevant studies were identified from each database, duplicate articles were removed. Studies were selected for inclusion in the review using a multistep screening strategy. First, studies that were identified through the search that was found to have an irrelevant title that did not include research questions relevant to the review were excluded. Next, we assessed the abstract and excluded those that did not fit with the research question. If the abstract indicated that the study collected data from a randomized controlled trial on a PA intervention for CRC survivors, the full text of the article was reviewed to determine the eligibility. To evaluate the suitability of the remaining studies, unrelated or nonapplicable studies were excluded by following inclusion and exclusion criteria. Two authors (Y.J. and H.S.) independently reviewed the identified articles to determine if they met all the inclusion criteria. Disagreements between the authors were resolved through discussion or consultation with a third author (J.C.) when it was necessary.

### Risk of Bias

Quality assessment was conducted using the Cochrane’s risk-of-bias tool that covers 6 domains of bias, including selection, performance, detection, attrition, reporting, and other bias.^23^ For the assessment of performance bias, it is generally difficult to blind participants in interventions targeting behavioral change because self-reported questionnaires are typically used to measure behavioral outcomes. Therefore, we did not assess the blinding of participants in our review. Two authors (Y.J. and H.S.) independently evaluated the risk of bias in each domain by classifying the risk as being *low*, *high*, or *unclear*. Disagreements between the authors were resolved through discussion until consensus was achieved or through consultation with another author (J.C.).

### Statistical Analysis

Meta-analysis was conducted using the Review Manager version 5.3 software.^30^ The weighted mean differences (MDs) or standardized mean differences (SMD) with 95% confidence intervals (CIs) were calculated for continuous outcomes. The statistical heterogeneity was quantified using the *I*^2^ statistic, which ranges from 0% to 100% and represents the proportion of interstudy variability that may contribute to the heterogeneity of the results rather than them being by chance. When *I*^2^ >50%, heterogeneity was determined to exist among the studies, and the random-effects model (DerSimonian-Laird method) was conducted for meta-analysis.^30^ We were unable to perform a subgroup analysis or sensitivity analysis because of an insufficient number of selected studies.

## Results

### Search Results

**Figure 1:**
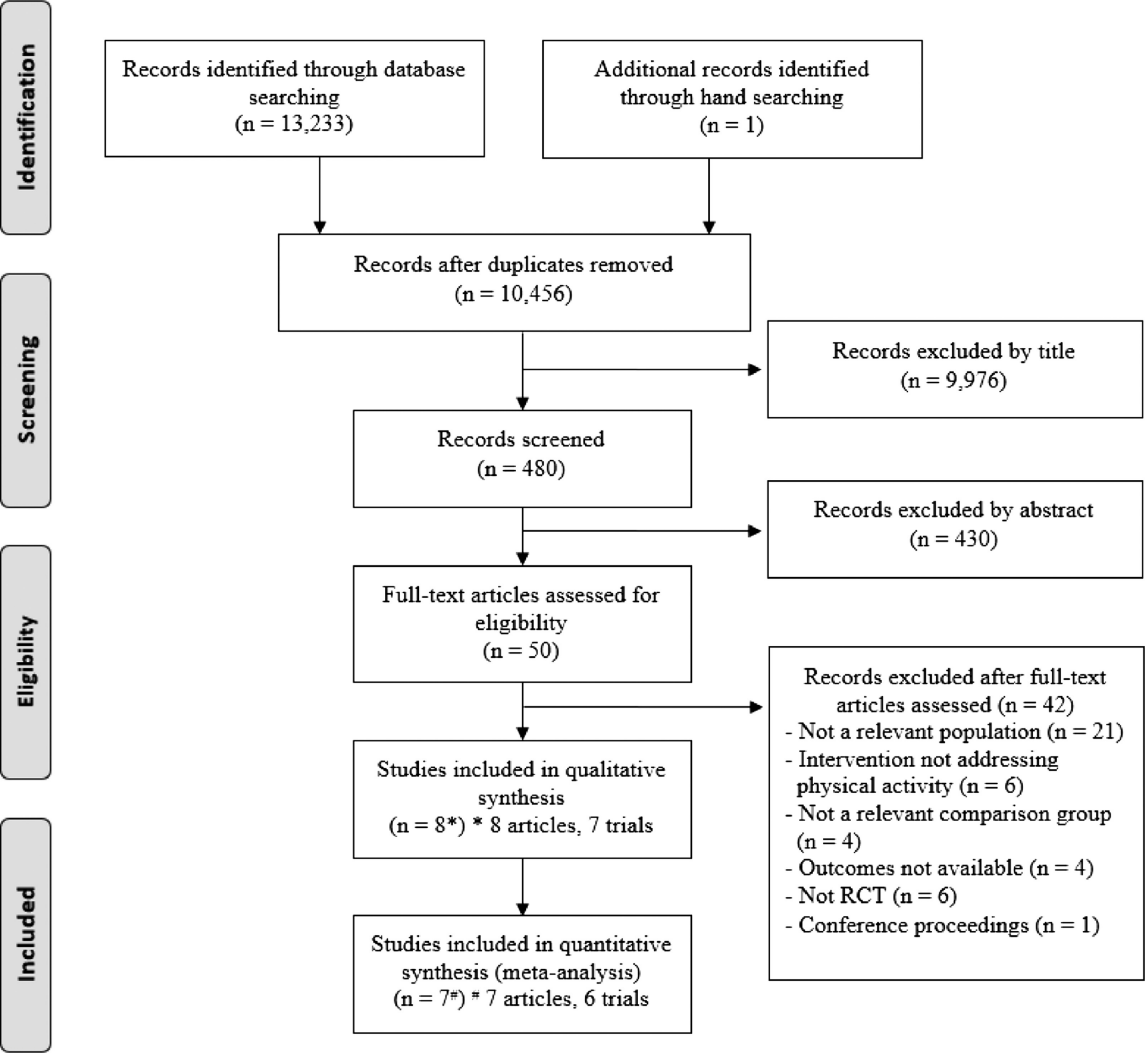
Flow diagram of the study selection process.

The electronic search yielded 13 233 potential studies, with one study being identified through a manual search. After duplicating records were removed, 10 456 articles remained. Further evaluation of titles and abstracts indicated that 10 406 did not meet the inclusion criteria. Of these, 50 full-text articles were obtained for detailed eligibility assessment. Forty-two articles that did not meet the eligibility criteria were excluded. Finally, 8 articles, including 7 trials, were selected for the systematic review. Of these, 2 articles^33,34^ were from the same trial. Consequently, 7 articles, including 6 trials, were used for the quantitative synthesis of meta-analysis. Figure 1 illustrates the article selection process.

### Risk of Bias in Included Studies

**Figure 2:**
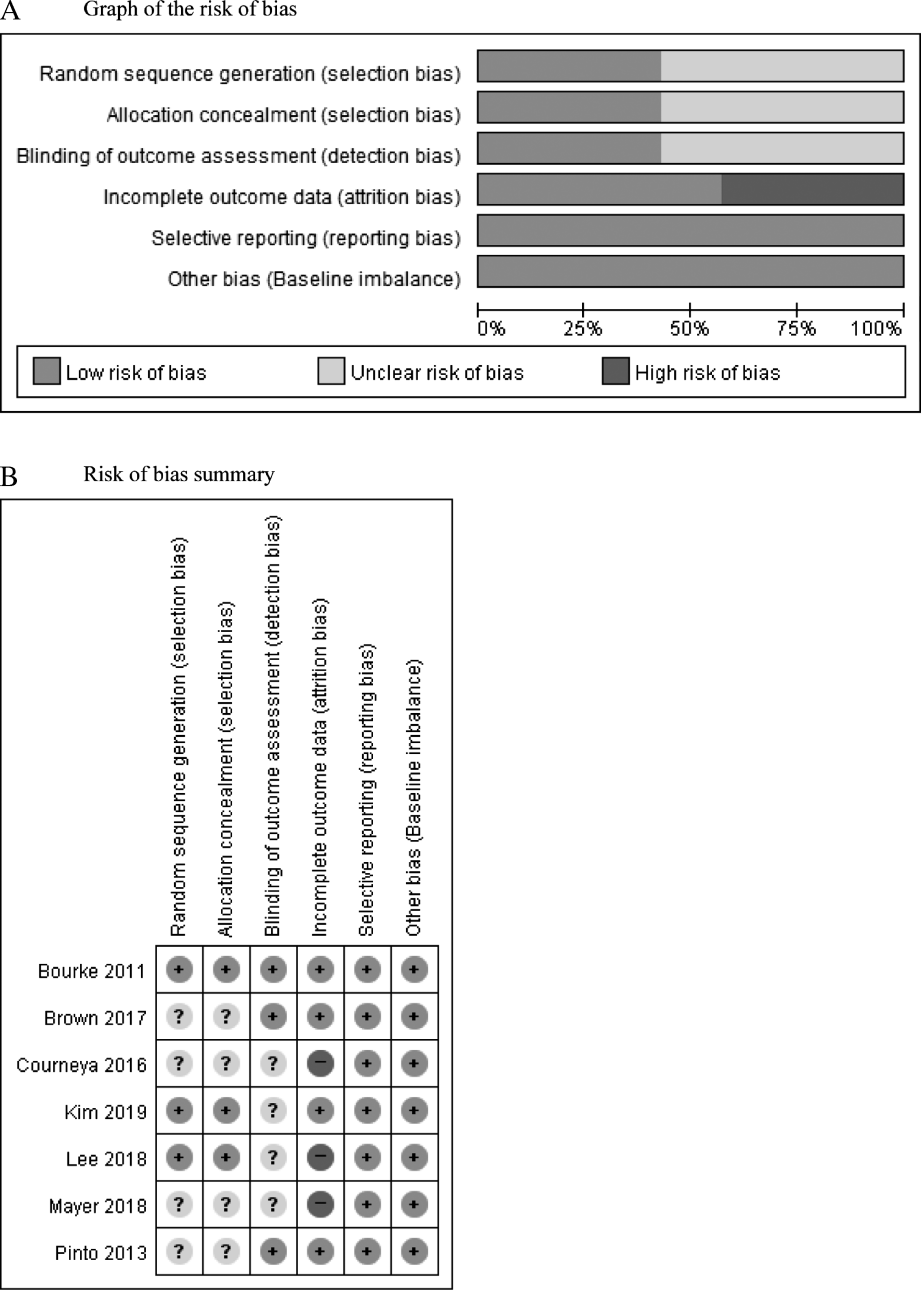
Assessment of the risk of bias in the final selection of included studies. A, Graph of the risk of bias. B, Risk-of-bias summary.

A summary of the overall risk of bias is presented in Figure 2. Three studies had a low risk of bias in selection (ie, random sequence generation and allocation concealment) and detection bias (ie, blinding of outcome assessment), whereas the other 4 studies had an unclear risk of bias. Three studies^3–35^ were evaluated as having a high risk of attrition bias as the dropout rates for these studies were greater than 20%. We also assessed the risk of reporting bias and other bias (baseline imbalance) and found that all studies were evaluated as low risk.

### Study Characteristics

The characteristics of the 7 selected articles are shown in Table 1. Three studies were conducted in the United States,^33,34,37,39^ whereas 2 studies were performed in South Korea.^35,36^ The remaining studies were carried out in the United Kingdom^40^ and in Canada and Australia.^38^ All of the studies were published after 2011. A total of 803 CRC survivors were included in the 7 studies, with the sample size of each ranging from 18 to 284 participants. Age and the proportion of women ranged from 56.2 to 69.0 years and 33.3% to 61.5%, respectively. In most studies, the description of the intervention delivered to the control group was not fully explained and was identified as being only “usual care.” Usual care could have included providing educational materials that addressed topics related to PA, symptom monitoring, or brief advice regarding the maintenance of usual behavior patterns.

**Table 1:** Characteristics of the Randomized Controlled Trials Included in the Review

Author Year Country	No. of Groups Sample Size Dropout Mean Age (SD) % Female	Cancer Stage Current Treatment	Intervention	Control	Outcome (1) Disease-Specific QoL (2) Level of Physical Activity (3) Fatigue (4) Maximum Amount of O_2_ (5) Body Mass Index	Results (1) Disease-Specific QoL (2) Level of Physical Activity (3) Fatigue (4) Maximum Amount of O_2_ (5) Body Mass Index
Kim et al^35^ (2019), South Korea	2 groups n = 71 (I: 37, C: 34) Dropout: 18.3% (n = 13) 56.2 y (SD, 9.4) 50.7% women	Stage II-III colorectal cancer survivors Completed all standard surgery and adjuvant chemotherapy within 4 wk to 2 y before the study enrollment	Home-based exercise program	Usual activities	(1) FACT-C (2) GLTPAQ (3) FACIT-FS (4) NA (5) NA	(1) No group differences (2) Significant improvement in intervention group (3) No group differences (4) NA (5) NA
Lee et al^36^ (2018), South Korea	2 groups n = 72 (I: 38, C: 34) Dropout: 20.8% (n = 15) 56.3 y (SD, 9.4) 51.4% women	Histologically confirmed stage II to III colorectal cancer survivors Colorectal cancer with completed surgery, radiotherapy, and/or chemotherapy within 4 wk to 2 y prior to study enrollment	Home-based mixed aerobic and resistance exercise program	Usual care	(1) NA (2) GLTPAQ (3) NA (4) NA (5) BMI	(1) NA (2) Significant increase in intervention group (3) NA (4) NA (5) No group difference
Mayer et al^37^ (2018), USA	2 groups n = 284 (I: 144, C: 140) Dropout: 20.1% (n = 57) I: 59.3 y (SD, 13.7), C: 57.8 y (SD, 14.5) 51.8% women	Pathologically confirmed Stage I-III colon cancer survivors Colon cancer with completed cancer treatment and at least 6 wk postoperative to within 12 mo of the diagnosis with no sign of recurrence	eHealth intervention - Smartphone with the survivor CHESS application - Intervention group received all materials provided to the control group	Usual care - Booklet - Audio program - Pedometer	(1) FACT-C 2) GLTPAQ (3) NA (4) NA (5) NA	(1) No group differences (2) No group differences (3) NA (4) NA (5) NA
Brown et al^33,34^ (2017, 2018), USA	3 groups n = 39 (low-dose: 14, high-dose: 12, C: 13) Dropout: 2.6% (n = 1) 56.5 y (SD, 10.0) s 61.5% women	Histologically-proven Stage I-III colon cancer survivors Completed cancer treatment(s) within 36 mo of entering the study	Aerobic exercise - Low-dose aerobic exercise - High-dose aerobic exercise	Usual care	(1) FACT-C (2) NA (3) FSI (4) NA (5) BMI	(1) Significant improvement in intervention group (2) NA (3) No group differences (4) NA (5) No significant change
Courneya et al^38^ (2016), Canada, Australia	2 groups n = 273 (I: 136, C: 137) Dropout: 22.7% (n = 62) I: 59 y, C: 61 y (median) 53.8% women	Stage II-III colon cancer survivors Received adjuvant chemotherapy within the past 2-6 mo	Structured exercise program	Health education materials - General health education materials promoting PA and healthy nutrition, standard surveillance follow-up	(1) NA (2) TPAQ (3) NA (4) Maximal oxygen uptake (Vo_2max_) (5) NA	(1) NA (2) Significant increase in intervention group (3) NA (4) No group differences (5) NA
Pinto et al^39^ (2013), USA	2 groups n = 46 (I: 20, C: 26) Dropout: 8.7% (n = 4) I: 59.5 y (SD, 11.2), C: 55.6 y (SD, 8.24) 56.5% women	Colon or rectal cancer (stages 1-3) Completed primary and adjuvant treatments ≤5 y since treatment completion	Home-based physical activity intervention	Contact control group - weekly calls to monitor symptom, received CRC survivorship tip sheets	(1) FACT-C (2) 7-d PAR (3) FACT-F (4) Maximal oxygen uptake (VO_2peax_) (5) NA	(1) No group differences (2) Significant increase in intervention group (3) No group differences (4) Significant increase in intervention group (5) NA
Bourke et al^40^ (2011), UK	2 groups n = 18 (I: 9, C: 9) Dropout: 5.6% (n = 1) 69 y (range: 52–80 y) 33.3% female	Colon cancer (Duke Stages A-C) 6-24 mo after primary treatment	Lifestyle intervention - Exercise - Diet	Standard care	(1) FACT-C (2) GLTPAQ (3) FACT-F (4) NA (5) BMI	(1) No group differences (2) No group differences (3) Significant improvement in intervention group (4) NA (5) No group difference

Abbreviations: BMI, body mass index; C, control; FACT-C, Functional Assessment of Cancer Therapy–Colorectal; FACIT-FS, Functional Assessment of Chronic Illness Therapy–Fatigue Scale; FCRI, Fear of Cancer Recurrence Inventory; FSI, Fatigue Symptom Inventory; GLTPAQ, Godin Leisure-Time Physical Activity Questionnaire; I, intervention; NA, not applicable; QoL, quality of life; TPAQ, Total Physical Activity Questionnaire; 7-day PAR, 7-day Physical Activity Recall.

Four studies included patients with colon cancer,^33,34,37,38,40^ whereas 3 studies involved patients with CRC.^35,36,39^ The cancer stage of the participants was from I to III in all studies. The elapsed time since their completion of primary treatment varied between the patients. The studies included patients who had completed cancer treatment within 5 years,^39^ since cancer treatment completion within 3 years,^33,34^ or within 2 years.^35,36,40^ Additionally, in 2 studies, patients were included within 2 to 6 months of receiving adjuvant chemotherapy^38^ or at least 6 weeks after surgery.^37^

The interventions for PA were also diverse. Six studies^33–39^ included a program targeting only PA, whereas 1 study^40^ provided an intervention targeting both PA and nutrition. All of the studies used home-based PA interventions, although 2 studies^38,40^ added supervised exercise sessions to the home-based PA program. A single study used an eHealth intervention with a smartphone.^37^

For the intervention periods, 3 studies conducted 12-week interventions,^35,39,40^ 2 lasted 6 months,^33,34,37^ and 1 study had a 6-week intervention.^36^ Finally, 1 study^38^ involved an intervention with a total duration of 3 years, but only reported the results from the first year of the intervention.

All studies employed a combination of methods of behavioral change. The most commonly used techniques were the self-monitoring of behavior, goal setting for the desired behavior, feedback, behavioral information, and social support (see Table 2 for details). Finally, the types of PA that were primarily used included aerobic exercise, such as brisk walking and biking, and a combination of resistance exercises.^35,36,40^

**Table 2:** Interventions of the Included Randomized Controlled Trials

Author Year	Intervention Intervention Details	Intervention Duration	Physical Activity Details				
			Exercise Type	Frequency	Intensity	Time	Progress
Kim et al^35^ (2019)	Home-based exercise program - Resistance exercise DVDs, a pedometer, an exercise log - Weekly phone counseling or small group training sessions - Daily test messages to check whether patients completed their daily exercise	12 wk	Aerobic exercises (unsupervised walking, stationary bike, or swimming) Resistance exercises	Daily	Moderate to vigorous intensity (18 METs to 27 METs)	NR	Exercise intensity was increased from 18 METs during the first 6 wk to 27 METs depending on individual health conditions
Lee et al^36^ (2018)	Home-based mixed aerobic and resistance exercise program - Exercise videos, exercise diary, a pedometer - Daily text message to increase compliance - Telephone counseling	6 wk	Aerobic exercises (brisk walking, hiking, stationary bike riding) Resistance exercises	Aerobic exercises: NR Resistance exercises: daily	≥18 METs	NR	NR
Mayer et al^37^ (2018)	eHealth intervention (smartphone with the survivor CHESS application) - Physical activity logging - Goal setting to ensure self-monitoring - Social networking to provide social support - Offering information on physical activity and health	6 mo	NR	NR	NR	150 min/wk	NR
Brown et al^33,34^ (2017, 2018)	Aerobic exercise (low-dose, high-dose) - Heart rate monitoring during exercise session - Paper logs to record adherence - Weekly telephone - Email communications	6 mo	In-home treadmills	NR	Moderate-intensity (50%-70% of the age-predicted maximum heart rate)	Low-dose: 150 min/wk High-dose: 300 min/wk	NR
Courneya et al^38^ (2016)	Structured exercise program - Clear and challenging exercise goals - Some supervised exercise sessions - Free or low-cost access to a fitness facility - Frequent and ongoing contacts including some face-to-face sessions with qualified staff - Individual tailoring of the intervention - Written materials - Counseling - Pedometer	1 y (total intervention duration is 3 y, but this report is the 1-y feasibility results)	Aerobic physical activity	Participants are able to choose the frequency of aerobic exercise to meet the intervention goal	Moderate-to-vigorous intensity	Participants are able to choose the time of aerobic exercise to meet the intervention goal	Recreational aerobic PA increase at least 10 MET-hours/week from baseline in the first 6 mo and sustain this change for 3 y
Pinto et al^39^ (2013)	Home-based physical activity intervention - In-person instructions - Home logs to monitor, a pedometer - Weekly call to monitor - Counseling	12 wk	Aerobic activities (brisk walking, biking, or use of home exercise equipment)	At least 5 d/wk	NR	30 min/d	Frequency and time gradually increased over the 12 wk
Bourke et al^40^ (2011)	Lifestyle intervention - Exercise: supervised and home-based exercise, exercise log book - Diet: dietary advice information pack, healthy eating seminars	12 wk	Aerobic exercise Resistance exercise	3 d/wk	55% to 85% of age predicted maximum heart rate	30 min (aerobic exercise)	NR

Abbreviations: C, control; I, intervention; MET, metabolic equivalent task; NR, not reported; PA, physical activity.

### Effects of Interventions

#### DISEASE-SPECIFIC QUALITY OF LIFE

Disease-specific quality of life was reported in 5 studies,^34,35,37,39,40^ and 3 of these studies were available for quantitative synthesis. In the meta-analysis, PA intervention significantly improved the disease-specific quality of life as compared with usual care (MD, 3.74; 95% CI, 0.22-7.25). Overall, the heterogeneity of study results was high (*I*^2^ = 71%). In 2 studies, quantitative synthesis was not possible^37,40^; however, disease-specific quality of life was not significantly different between the 2 groups.

#### LEVEL OF PA

The level of PA was reported in 6 studies,^35–40^ and 3 of these studies were available for quantitative synthesis. The pooled data showed a significant rise in the level of PA for those receiving the intervention (SMD, 0.80; 95% CI, 0.28-1.32). Overall, heterogeneity of study results was high (*I*^2^ = 79%). One of the 3 studies for which quantitative synthesis was not possible reported a significant difference in the level of PA,^39^ whereas 2 studies^37,40^ reported no significant differences between the intervention and control groups.

#### FATIGUE

Fatigue was reported in 4 studies,^33,35,39,40^ and 3 of these studies were available for quantitative synthesis. In the meta-analysis, PA intervention did not show a significant effect for reducing participants’ fatigue (SMD, 0.17; 95% CI, −0.37 to 0.72; *I*^2^ = 45%). On the other hand, there was no significant effect on fatigue for the 1 study for which quantitative synthesis was not possible.^39^

#### THE MAXIMUM AMOUNT OF OXYGEN

The maximum amount of oxygen a person can utilize during PA^32^ was assessed using Vo_2max_ in 2 studies.^38,39^ The pooled effects showed a significant improvement in the intervention group as compared with usual care (MD, 3.19; 95% CI, 1.24-5.13; *I*^2^ = 0%).

#### BODY MASS INDEX

**Figure 3:**
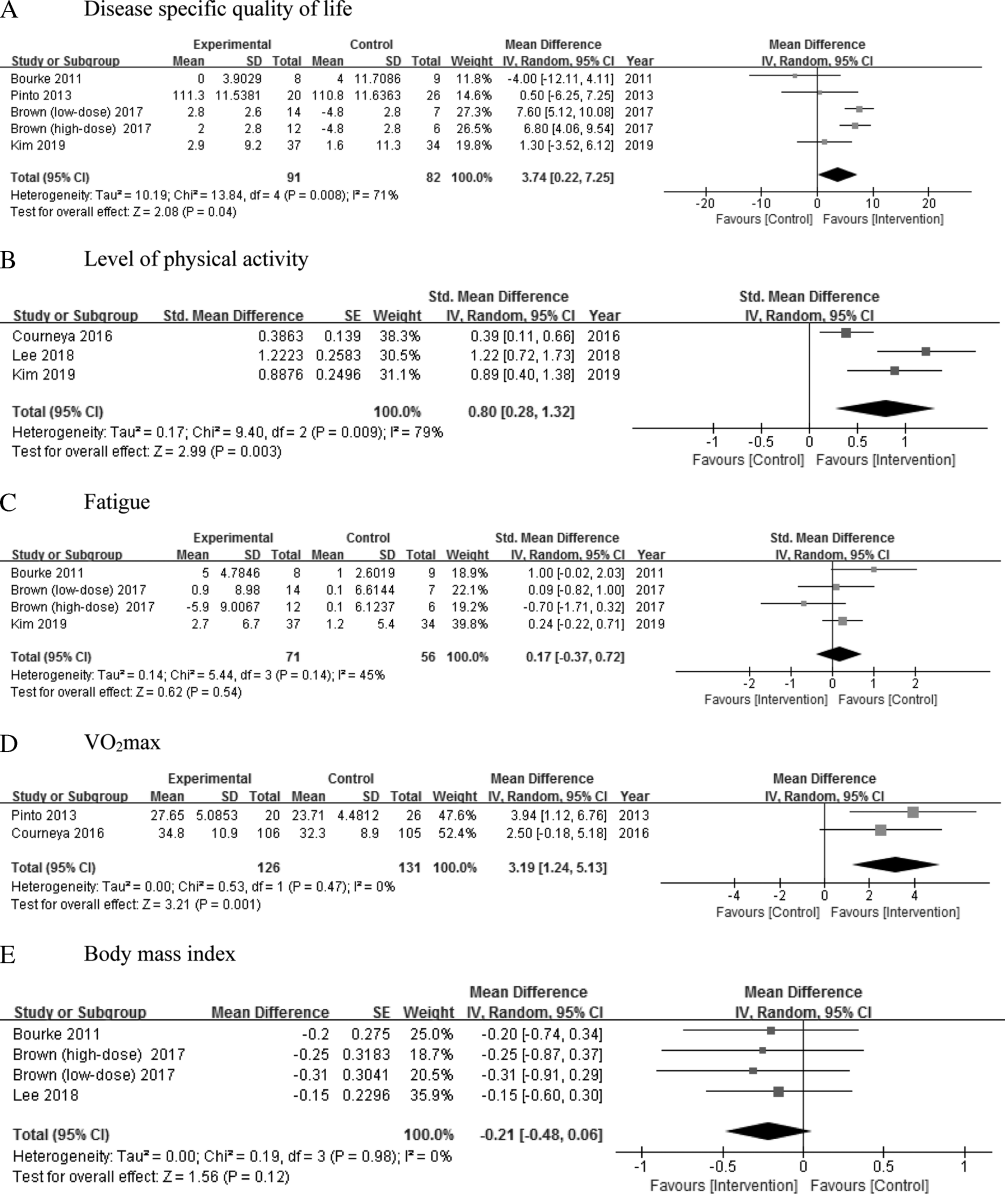
Forest plot of meta-analysis on effects of physical activity intervention. A, Disease-specific quality of life. B, Level of physical activity. C, Fatigue. D, Vo_2_max. E, Body mass index.

Body mass index was reported in 3 studies.^34,36,40^ Physical activity intervention was not found to significantly decrease BMI compared with usual care (MD, −0.21; 95% CI, −0.48 to 0.06; *I*^2^ = 0%) (See Figure 3 for all outcomes).

## Discussion

As the survival rate of CRC has improved, the survivors of CRC need to increase their PA when they return to their homes and community after completing primary treatment. More than three-fourths of all cancer survivors have reported supportive care needs, and their highest perceived needs have included healthy lifestyle programs,^41^ suggesting the need for increased attention on survivors during the posttreatment period. Previous studies, including systematic reviews and meta-analyses, have examined the effect of PA on the various types of cancer, but the unique effect of PA on CRC patients is still unclear. To the best of our knowledge, this study is the first to examine the effect of PA interventions on CRC survivors.

We found that PA interventions significantly improved disease-specific quality of life compared with those patients in the control group. The positive association between PA and quality of life for CRC survivors has previously been confirmed for various types of cancer survivors.^18,19,28^ For CRC survivors, particularly, prospective studies have suggested a relationship between PA and quality of life during the 2 years following the cancer diagnosis, with these associations being found both between and within participants.^42^ Furthermore, a dose-response relationship between PA and quality of life for CRC survivors has been found, which indicates that the more intense PA, the better quality of life.^43^ Although strong evidence supports the benefits of PA for CRC survivors’ quality of life, the mechanisms that explain why PA increases the quality of life have not been fully explained. A plausible explanation could be made for the context as considering the relationship between PA and quality of life. Lynch and colleagues^44^ described the association between sedentary behavior and quality of life, stating that sedentary behaviors, such as television viewing, may reduce social interaction and quality of life. During the PA intervention, the additional forms of social interaction (ie, face-to-face or remote coaching) could also contribute to the survivors’ increased quality of life, particularly the social and emotional aspects. Future investigations are needed to clarify the etiologic nature of the relationship between PA and quality of life among CRC survivors.

In our meta-analysis, we found that PA interventions did not significantly improve fatigue for CRC survivors. Previous reviews are consistent with this finding, indicating that PA interventions have not identified significant group differences in patients’ fatigue.^26,27^ Fatigue in cancer patients has been acknowledged as a multifactorial construct that encompasses both physical symptoms (ie, tiredness) and psychological disturbance, and research has suggested the presence of a complex interaction between various etiologic mechanisms.^45^ Therefore, PA interventions that focus on the physical aspect of a patient’s health and well-being may not contribute to improving fatigue, suggesting that multimodal interventions that combine PA with psychosocial approaches could be more appropriate to ameliorate fatigue in CRC survivors.

In the current study, BMI was shown to decrease in both the intervention and control groups with no significant differences for changes in BMI between the groups, which is consistent with a recent randomized controlled trial promoting PA in CRC and endometrial cancer survivors.^19^ In a 2-year longitudinal study,^42^ additionally, a change in BMI between 6 and 12 months following diagnosis was observed, but no significant interactions were found between PA, BMI, and quality of life. Lynch and colleagues^42^ discussed these results in the context of the timing of diagnosis and cancer treatment, indicating that most participants have typically returned to their previous BMI within 1 year after diagnosis. Because the participants in our study completed the primary treatment, the absence of significant findings may reflect this explanation. Furthermore, this pattern of findings may be explained by the discrete characteristics of the definition of PA as compared with exercise. The terms *PA* and *exercise* are used interchangeably; however, they are conceptually different. As a subset of PA, exercise is defined as a planned, structured, and repetitive form of PA, whereas the broader construct of PA is characterized as being any bodily movement made by skeletal muscles.^46^ As a complex behavior, PA encompasses light-, moderate-, or heavy-intensity physical activities.^46^ Thus, targeting the broader construct of PA in an intervention might be sufficient to change BMI only when the intervention has a longer duration (eg, >12 weeks), different modes, volumes, or intensities of PA.

Regarding the place and delivery method of the interventions, most of the included studies used home-based exercise, although 2 studies^38,40^ included supervised exercise sessions. A home-based study using current wearable technology, such as activity trackers, was adopted in many research settings to help cancer survivors self-assess their progress at home.^47^ Smartphone and web-based interventions have also been popularly used as an effective modality to improve PA^48,49^; a high need for eHealth for the supportive care of cancer survivors is needed.^41^ One study by Mayer and colleagues^37^ utilized a smartphone application and reported the lack of significant differences in the level of PA between intervention and control groups, although PA increased in both groups. The absence of significant differences might be related to the fact that the individuals in the control group also received a type of intervention, which led to the increased PA. They were provided with an educational booklet, audio program, and a pedometer, whereas the smartphone application was added only for those in the intervention group. Although previous research has reported the substantial impact of eHealth, further research is required to identify the effective method of delivering PA intervention for CRC survivors (ie, home-based, supervised, or internet-based).

In practice, a tailored PA intervention needs to be developed to improve disease-specific quality of life, the level of PA, and the maximum amount of oxygen. Based on our findings, specific considerations are necessary when developing a PA intervention for CRC survivors. We showed that the interventions significantly improved the level of PA and fatigue in 2 studies^38,40^ that included supervised exercise sessions. Another study also demonstrated that supervised training is more effective for PA than is unsupervised training.^50^ Because most interventions for survivors are conducted in the community, independently initiating PA after they are discharged from the hospital may be challenging. Therefore, the supervised sessions in the PA program are needed.

In addition, the intervention durations of the selected studies varied from 6 weeks to 3 years. Except for 1 study,^38^ most interventions had a relatively short duration lasting less than 6 months. The selected studies showed that the increased PA was not maintained over time when the intervention was complete.^37,39^ Evidence from the previous study supports that interventions with booster sessions improved long-term rehabilitation outcomes for cardiac and orthopedic patients.^51^ To make PA intervention more effective during a short period, therefore, a booster session could be used as a supplementary method after the intervention is provided.

As PA barriers differ by the types of cancer,^52^ CRC survivors more frequently encounter a number of disease-specific and treatment-related difficulties such as bowel dysfunction that contribute to physical inactivity.^53,54^ For CRC survivors, a simple recommendation may be insufficient to practice PA after they are discharged. Therefore, healthcare providers must develop a more personalized PA plan with the CRC survivors before they complete the primary treatment such as paying close attention to the potential barriers or perceived difficulties for PA that the survivors may confront.

### Study Limitations

There are several limitations to this meta-analysis. First, the number of studies included in the current study is relatively small. As the total sample size was also small, conducting subgroup analyses was not possible. Thus, we could not conclusively determine which types of PA interventions were effective for CRC survivors. Additional randomized controlled trial studies are required in the future. Second, the characteristics of CRC survivors were not comparable in each of the selected studies. The frequency, intensity, type, and duration of PA could be essential components when developing an effective intervention.^55^ Owing to the heterogeneity of the studies and the relatively small sample size included in the current meta-analysis, it was not possible to conduct a subgroup analysis to determine which characteristics are the most effective modality for a PA intervention. Finally, the duration of the interventions was relatively short. Because engaging in PA across their life span is essential for CRC survivors, additional randomized controlled trials with longitudinal designs are needed to establish the long-term effects of PA to understand the benefits of daily and self-directed PA.

## Conclusions

This systematic review and meta-analysis provided evidence that PA interventions were effective on disease-specific quality of life, the level of PA, and the maximum amount of oxygen, while the interventions did not improve fatigue and BMI. Additional randomized controlled trials are required to determine the specific optimal mode of delivering PA intervention for CRC survivors, as the effective and efficient mode of PA intervention delivery may improve health-related outcomes and specific barriers or difficulties in CRC survivors.

## Appendix 1. Search Terms

**Ovid MEDLINE**

1. exp Colectal Neoplasms/
2. ((colorectal$ or colon$ or rect$) adj2 (cancer$ or neoplasm$ or tumor$ or tumor$ or malignan$ or carcinoma$ or adenocarcinom$)).tw.
3. 1 or 2
4. exp Exercise/
5. exp Exercise Therapy/
6. exercis$.tw.
7. physical activit$.tw.
8. exp Life Style/
9. (lifestyle or life style).tw.
10. exp Sports/
11. walking/
12. running/
13. jogging/
14. bicycling/
15. swimming/
16. 4 or 5 or 6 or 7 or 8 or 9 or 10 or 11 or 12 or 13 or 14 or 15
17. 3 and 16
18. animal/
19. human/
20. 18 not 19
21. 17 not 20

**EMBASE**

1. ‘large intestine tumor’/exp
2. (colorectal* OR colon* OR rect*) NEAR/2 (cancer* OR neoplasm* OR tumor* OR tumor* OR malignan* OR carcinoma* OR adenocarcinom*)
3. #1 or $2
4. ‘exercise’/exp
5. ‘kinesiotherapy’/exp
6. exercis*
7. ‘physical activity’/exp OR ‘physical activit*’
8. ‘lifestyle modification’/exp
9. (lifestyle OR ‘life style’) NEAR/2 (modification* OR intervention* OR change*)
10. sport/exp
11. walking/exp
12. running/exp
13. jogging/exp
14. cycling/exp
15. swimming/exp
16. #4 or #5 or #6 or #7 or #8 or #9 or #10 or #11 or #12 or #13 or #14 or #15
17. #3 and #16
18. animal/exp
19. human/exp
20. #18 not #19
21. #17 not #20

**CENTRAL (Cochrane Central Register of Controlled Trials)**

1. MeSH descriptor: [Colorectal Neoplasms] explode all treescirrhosis
2. ((colorectal* or colon* or rect*) NEAR/2 (cancer* or neoplasm* or tumor* or tumor* or malignan* or carcinoma* or adenocarcinom*)):ti,ab
3. #1 or #2
4. MeSH descriptor: [Exercise] explode all trees
5. MeSH descriptor: [Exercise Therapy] explode all trees
6. exercis*:ti,ab
7. physical activit*:ti,ab
8. MeSH descriptor: [Life Style] explode all trees
9. (lifestyle or life style):ti,ab
10. MeSH descriptor: [Sports] explode all trees
11. (walk* or run* or jog* or bicycl* or cycl* or swim*):ti,ab
12. #4 or #5 or #6 or #7 or #8 or #9 or #10 or #11
13. #3 and #12

**CINAHL (Cumulative Index to Nursing and Allied Health Literature)**

1. MH “Colorectal Neoplasms+”
2. TX ((colorectal* OR colon* OR rect*) N2 (cancer* OR neoplasm* OR tumor* OR tumor* OR malignan* OR carcinoma* OR adenocarcinom*))
3. S1 OR S2
4. MH “Exercise+”
5. MH “Therapeutic Exercise+”
6. TX exercis*
7. TX “physical activit*”
8. MH “Life Style+”
9. MH “Life Style+”
10. MH “Sports+”
11. TX (walk* or run* or jog* or bicycl* or cycl* or swim*)
12. S4 OR S5 OR S6 OR S7 OR S8 OR S9 OR S10 OR S11
13. MH “experimental studies+”
14. TI (randomized or randomized or placebo or randomly or trial or “quasi-experiment*” or pre test or pretest or posttest or “post test” or “time series” or “controlled stud*” or “before and after” or “controlled before”)
15. AB (randomized or randomized or placebo or randomly or trial or “quasi-experiment*” or pre test or pretest or posttest or “post test” or “time series” or “controlled stud*” or “before and after” or “controlled before”)
16. S13 OR S14 OR S15
17. S3 AND S12 AND S16
